# Supplementary material for: India’s potential for integrating solar and on- and offshore wind power into its energy system
Source: Nat Commun. 2020 Sep 21;11:4750. doi: 10.1038/s41467-020-18318-7 (PMC7505833; doi:10.1038/s41467-020-18318-7)
Supplement: Supplementary file 1 — Supplementary Information [file 41467_2020_18318_MOESM1_ESM.pdf]

## Supplemental Information

# India's potential for integrating solar and on- and offshore wind power into its energy system

Tianguang Lu, Peter Sherman, Xinyu Chen, Shi Chen, Xi Lu, Michael McElroy

**Supplementary Table 1** Specifications for the on- and offshore turbines used here.

| Specifications | GE 2.5 MW | Vestas 8.0 MW |
|---|---|---|
| Rated power | 2,500.0 kW | 8,000.0 kW |
| Cut-in wind speed | 3.0 m/s | 4.0 m/s |
| Rated wind speed | 13.0 m/s | 13.0 m/s |
| Cut-out wind speed | 25.0 m/s | 25.0 m/s |
| Diameter | 100.0 m | 164.0 m |
| Swept area | 7,853.9 $m^2$ | 21,164.1 $m^2$ |
| Number of blades | 3 | 3 |

**Supplementary Table 2.** Per unit costs [$/kW] associated with interregional transmission of power.

| Interregional corridor | Cost [$/kW] |
|---|---|
| East-North | 65.21 |
| East-Northeast | 48.19 |
| East-South | 61.72 |
| East-West | 2.07 |
| North-West | 63.85 |
| North-Northeast | 48.19 |
| South-West | 107.46 |

**Supplementary Table 3.** Costs for mechanical and chemical storage associated with the three scenarios. E represents the energy-specific cost in $/kWh and P represents the power-specific cost in $/kW.

| | Low cost | | Medium cost | | High cost | |
|---|---|---|---|---|---|---|
| | E | P | E | P | E | P |
| Mechanical Storage | 60 | 1200 | 70 | 1400 | 80 | 1600 |
| Chemical Storage | 120 | 240 | 160 | 320 | 200 | 400 |

**Supplementary Table 4.** Properties of small (10-300 MW), medium (300-600 MW) and large (600-1000 MW) thermal units (coal and natural gas). Data from the IEA [1].

| | Coal | | | Gas | | |
|---|---|---|---|---|---|---|
| | Small | Medium | Large | Small | Medium | Large |
| Max. output [%] | 100 | 100 | 100 | 100 | 100 | 100 |
| Min. output [%] | 50 | 50 | 50 | 25 | 40 | 40 |
| Capital cost [$/kW] | 621 | 585 | 515 | 524 | 488 | 418 |
| O&M [% of Capital cost] | 2.1 | 2.0 | 1.9 | 2.6 | 2.5 | 2.4 |
| Start-up cost [$/MW] | 147 | 147 | 147 | 88 | 88 | 88 |
| Min. up time [hr.] | 8 | 8 | 24 | 1 | 4 | 6 |
| Min. down time [hr.] | 4 | 8 | 48 | 1 | 2 | 12 |
| Ramping limit [%/hr.] | 35 | 35 | 35 | 100 | 50 | 50 |

**Supplementary Table 5.** Inputs for the cost-optimization model. Columns indicate the different scenarios investigated in the cost-optimization model, specifically (1) the standard scenario (standard), (2) the standard scenario with low cost storage (low cost storage), (3) the standard scenario with no storage (no storage), (4) the standard scenario with high cost renewables (high cost renewables) and (5) the standard scenario with no storage and high price gas (high gas price).

| | Standard | Low cost storage | No storage | High cost renewables | High gas price |
|---|---|---|---|---|---|
| PV [$/kW] | 550 | 550 | 550 | 1650 | 550 |
| Onshore wind [$/kW] | 980 | 980 | 980 | 1950 | 980 |
| Offshore wind [$/kW] | 1300 | 1300 | 1300 | 2300 | 1300 |
| Coal fuel [$/MMBTU] | 3.6 | 3.6 | 3.6 | 3.6 | 3.6 |
| Gas fuel [$/MMBTU] | 7.65 | 7.65 | 7.65 | 7.65 | 11 |
| Coal and gas options | Table S3 | Table S3 | Table S3 | Table S3 | Table S3 |
| Transmission | Table S1 | Table S1 | Table S1 | Table S1 | Table S1 |
| Storage | Medium cost, Table S2 | Low cost, Table S2 | -- | Medium cost, Table S2 | -- |

**Supplementary Table 6.** Parameter settings for the solar filter.

| Parameters | | Setting |
|---|---|---|
| **Suitability Factor of Land use (%)** | All forest | 0 |
| | Closed shrublands, woody savannas | 5 |
| | Grasslands, open shrublands, savannas | 20 |
| | Barren or sparsely vegetated | 20 |
| | Croplands, cropland/natural vegetation mosaic | 0 |
| | Urban and built-up | 5 |
| | Water bodies, permanent wetlands, snow and ice | 0 |
| **Slope (%)** | | < 5 |
| **Solar Radiation (kWh/(m$^2$·a))** | | > 1400 |

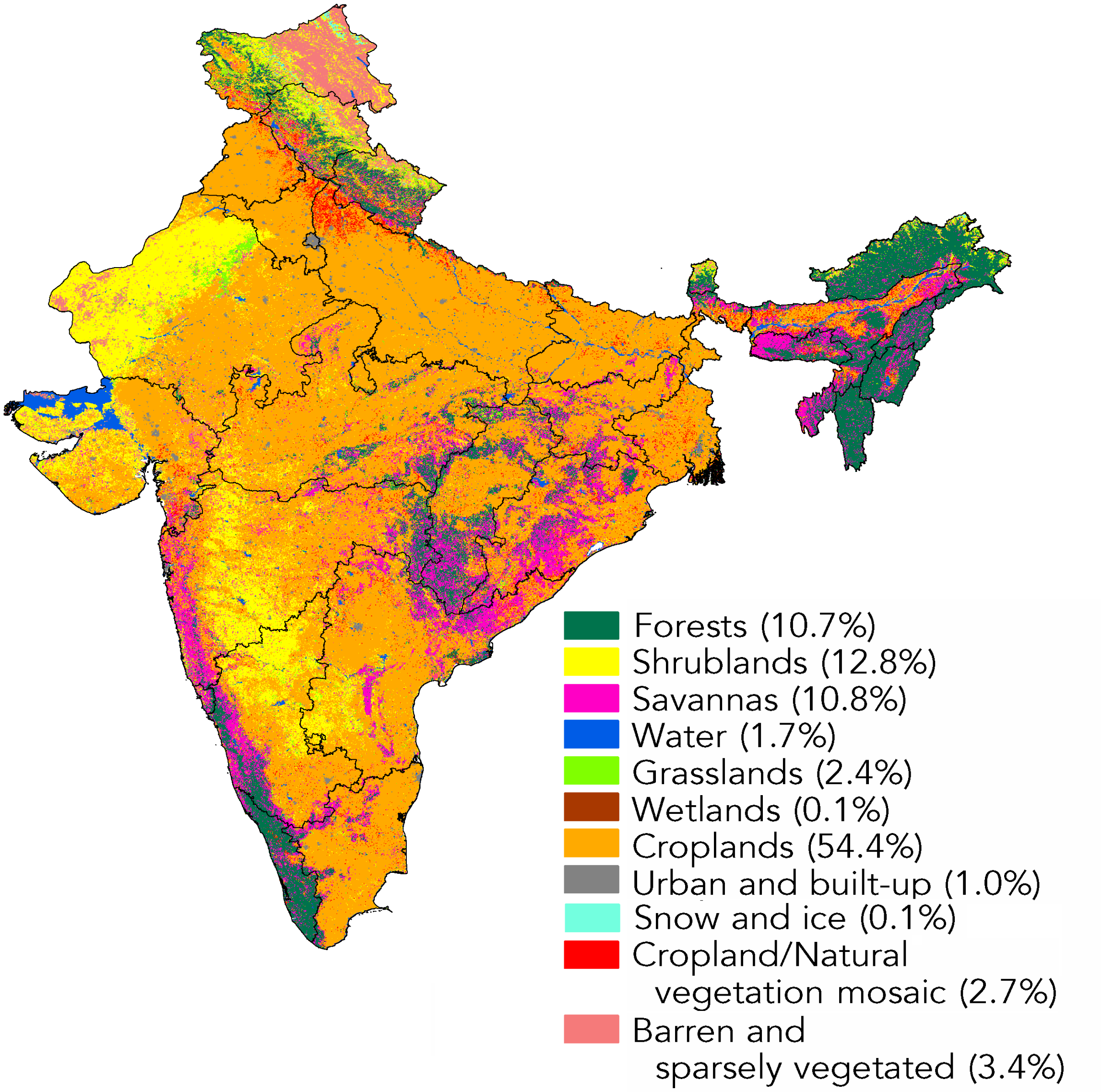


**Supplementary Figure 1 Land type classifications.** Classification of different land types as determined by the MODIS (Moderate Resolution Imaging Spectroradiometer) satellite MCD12C1 dataset [2]. The percentage of India's overall land mass classified as a specific land type is denoted in parenthesis in the legend. This figure was constructed in MATLAB version 2017b and edited in Adobe InDesign 2020.

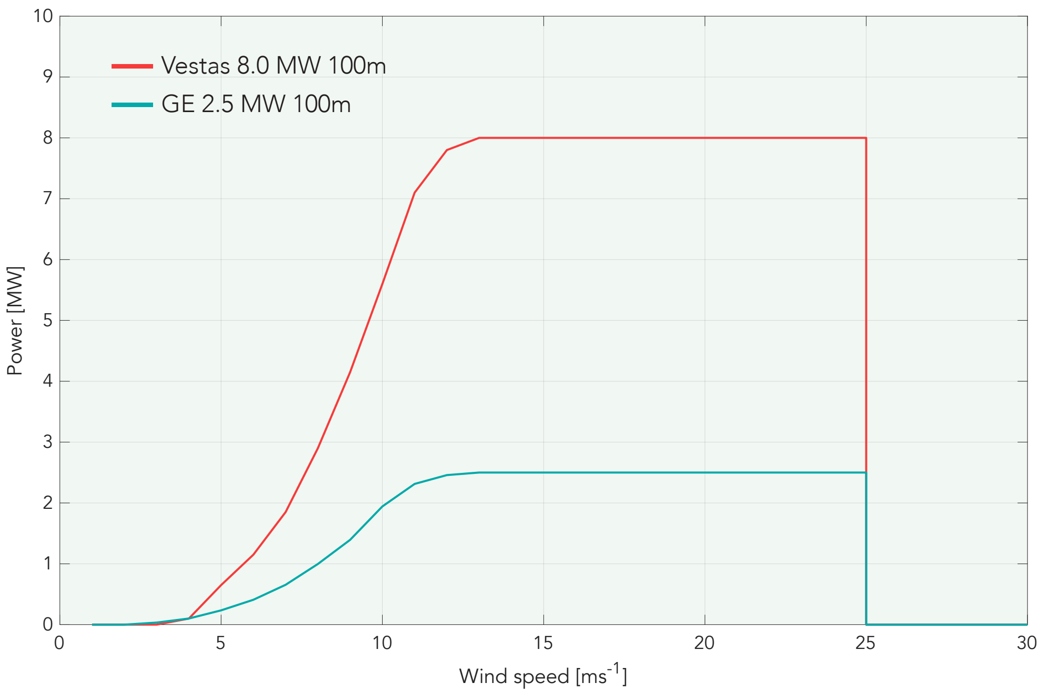


**Supplementary Figure 2 Wind power curves.** Wind power curves for the two wind turbines used in this study.

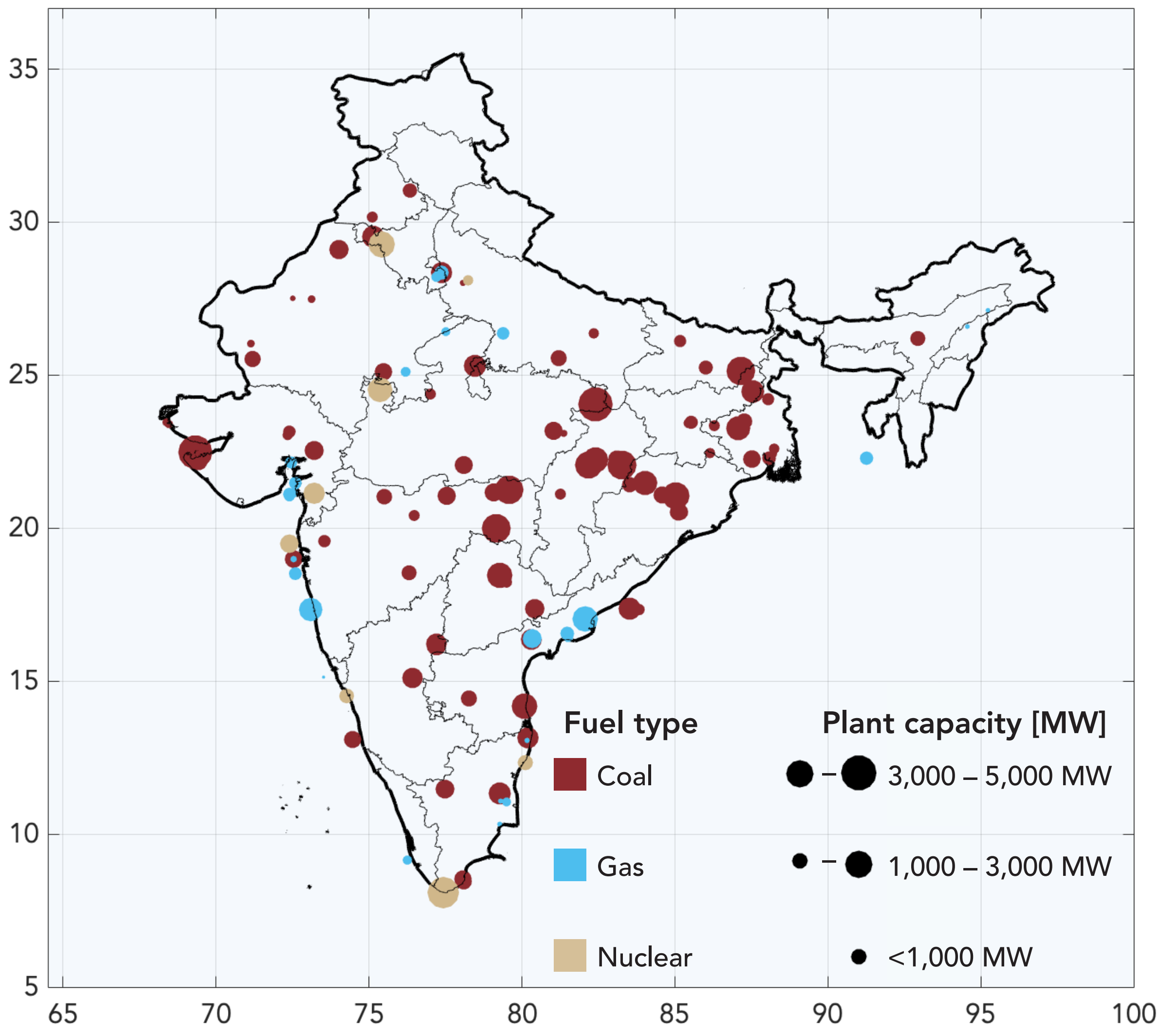


**Supplementary Figure 3 Geographical distribution and capacities for coal, gas and nuclear power stations.** Coal plants are indicated in dark red, gas in blue and nuclear in light brown. Coal data are derived from [3] and gas and nuclear data are derived from [4]. This figure was constructed in MATLAB version 2017b and edited in Adobe InDesign 2020.

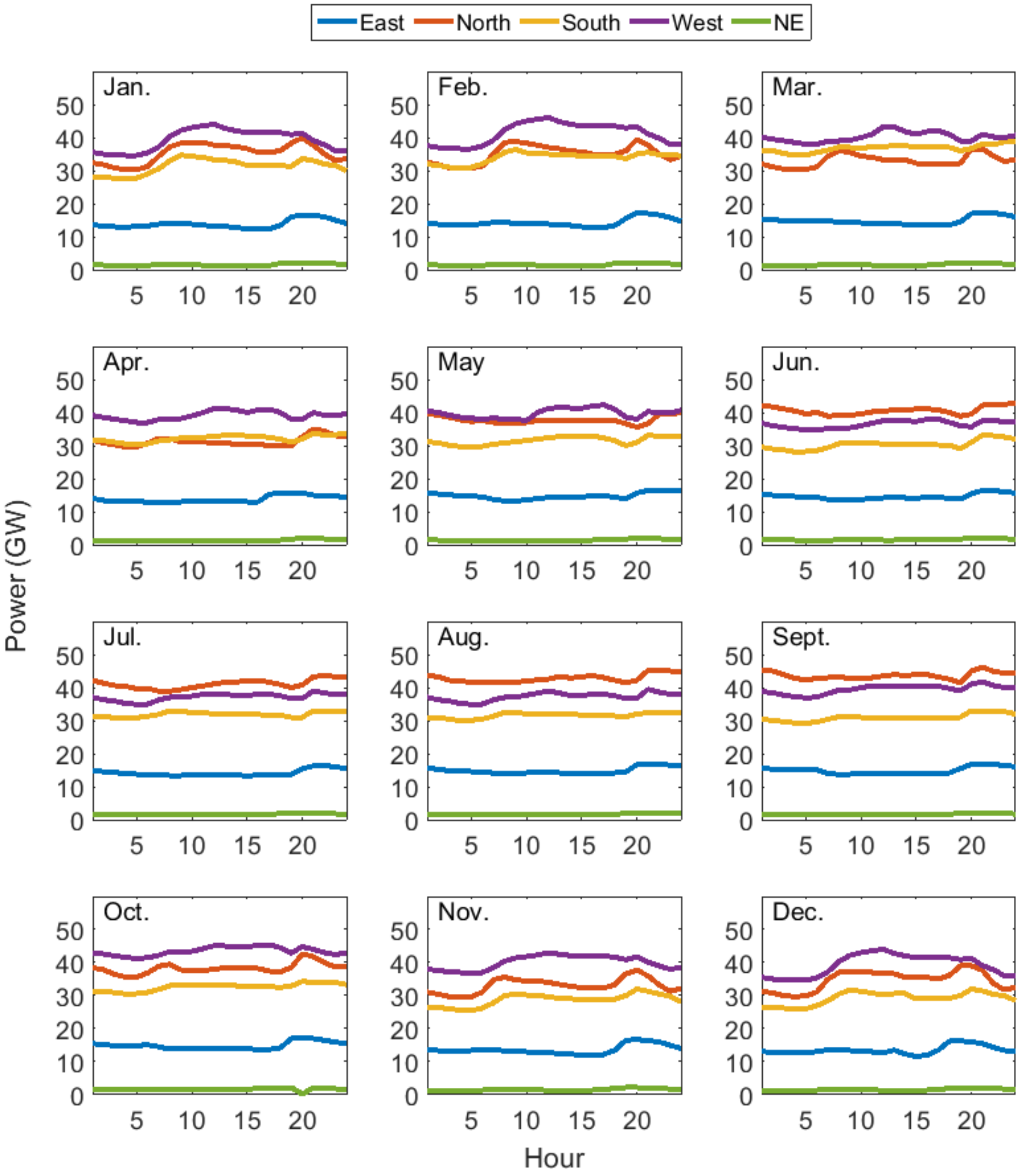


**Supplementary Figure 4 Hourly power demand in 2016.** Hourly data for five regions for a typical day in each month derived from POSOCO [5].

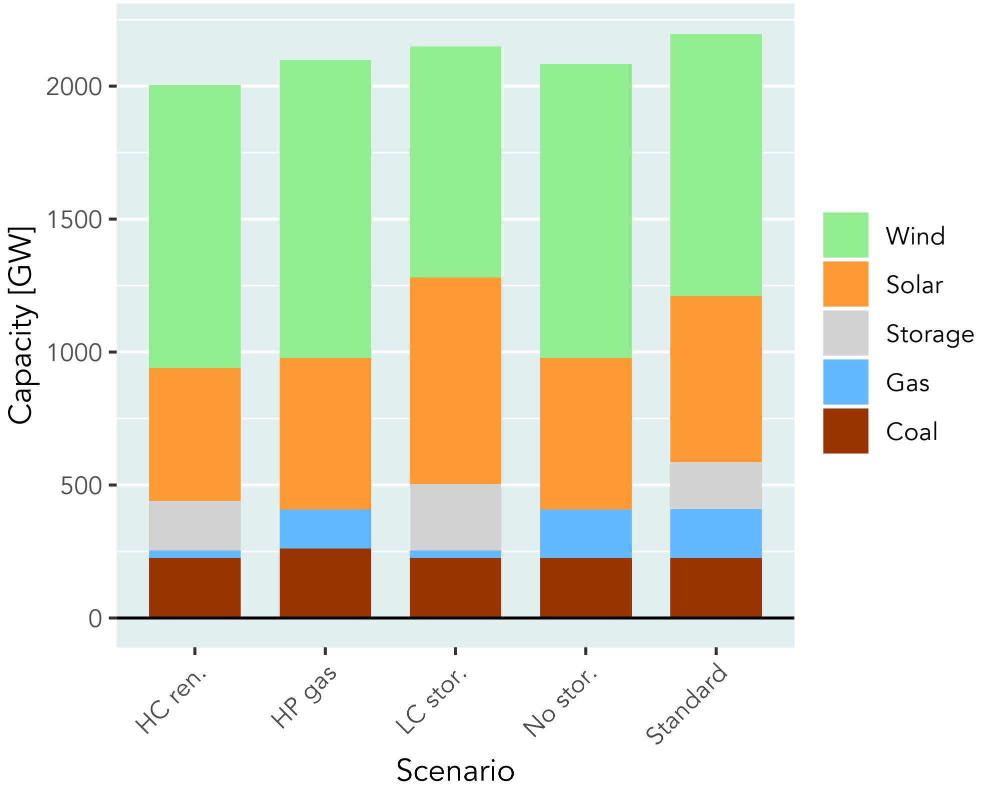


**Supplementary Figure 5 National generation mix.** National generation mix of capacities (GW) for 80% renewables as a function of different scenarios summarized in Supplementary Table 5.

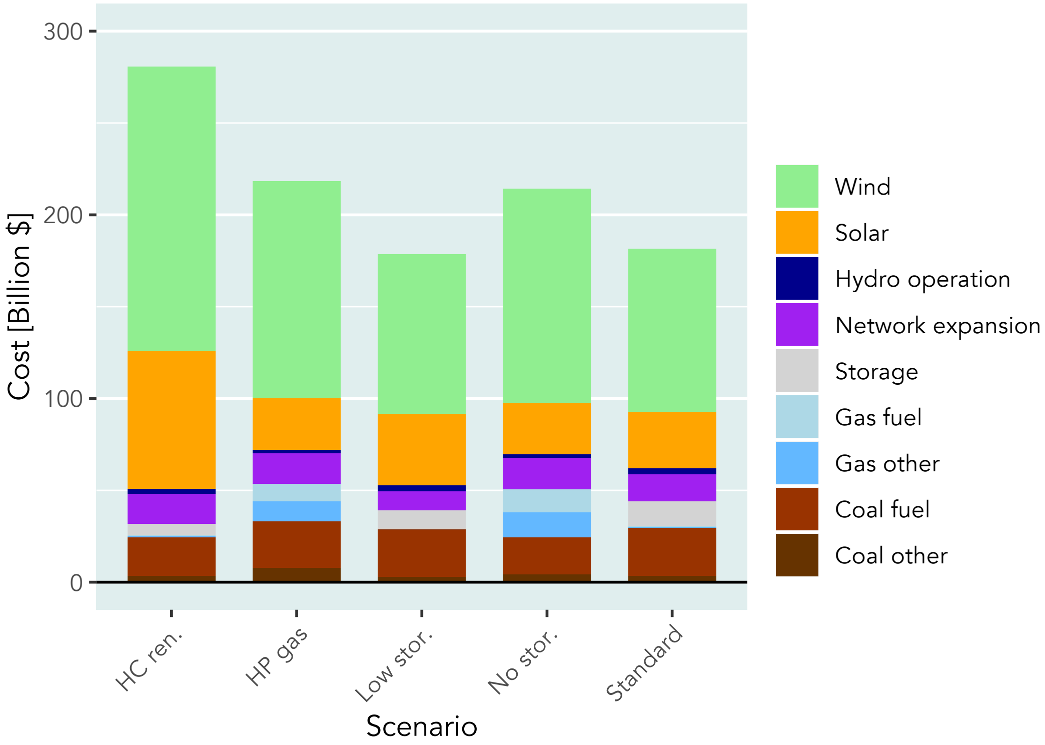


**Supplementary Figure 6 National system costs.** System costs for 80% renewables as a function of different scenarios summarized in Supplementary Table 5.

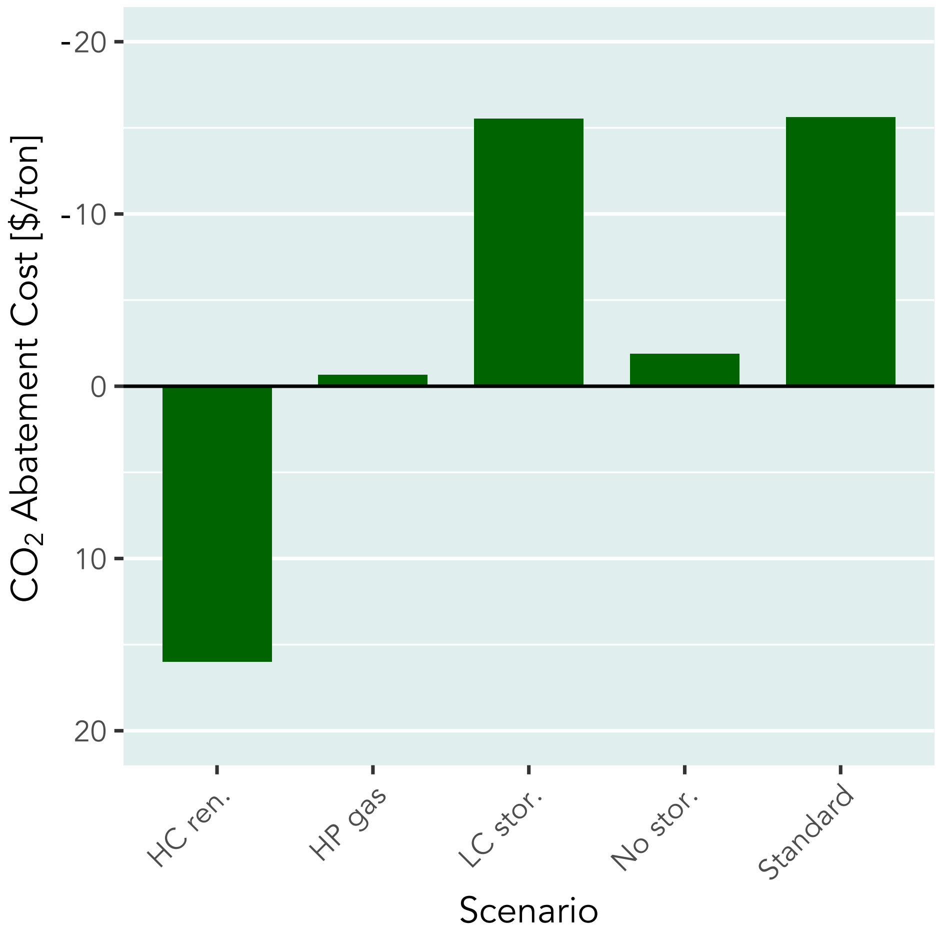


**Supplementary Figure 7 $CO_2$ reduction costs.** Costs for $CO_2$ reductions [in $/ton] for 80% renewables as a function of different scenarios summarized in Supplementary Table 5.

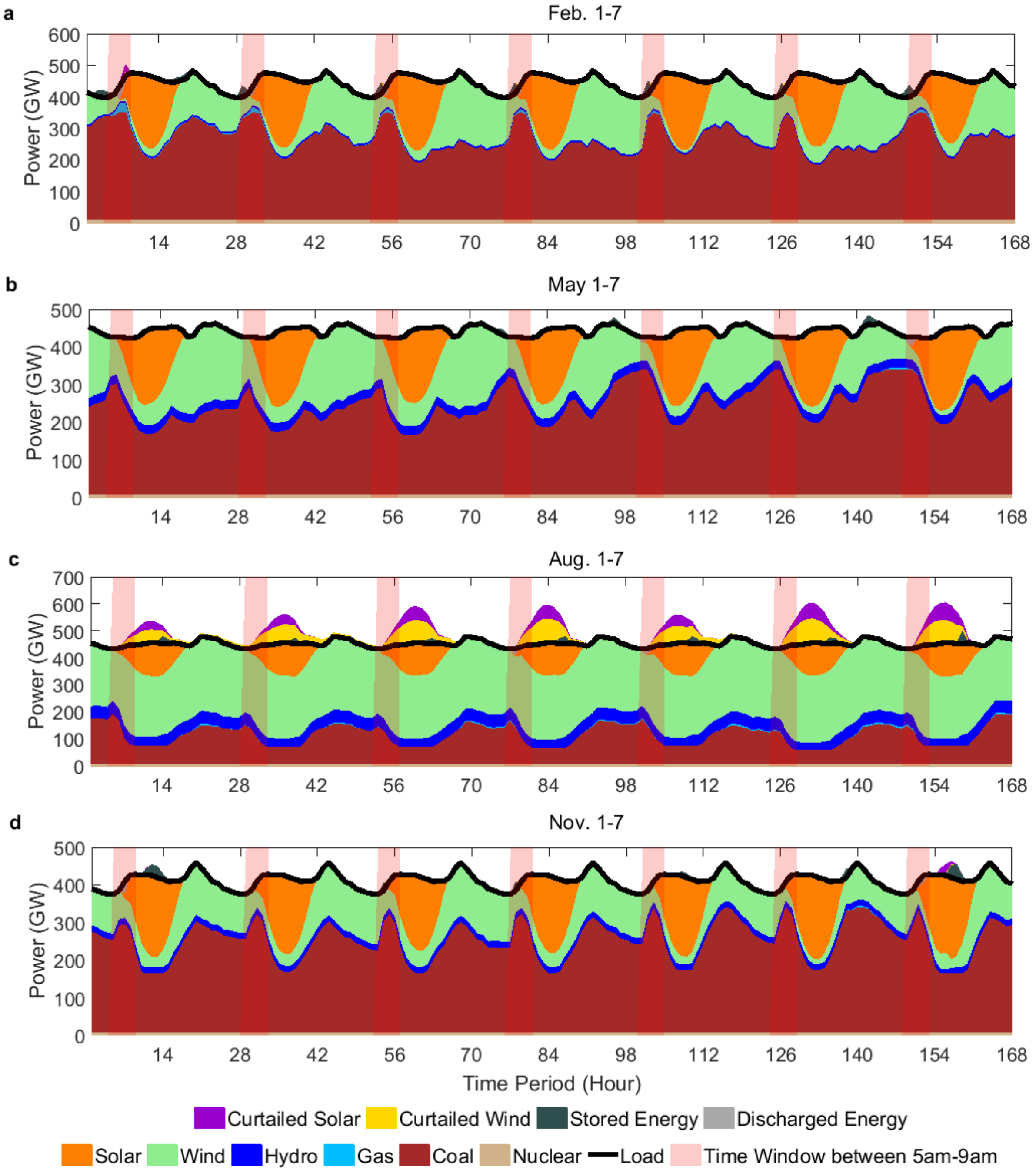


**Supplementary Figure 8 Seasonal hourly power balance.** Hourly power balances defined for seasonally representative weeks on the basis of the 40% renewables standard model simulation: February 1-7 (a), May 1-7 (b), August 1-7 (c) and November 1-7 (d).

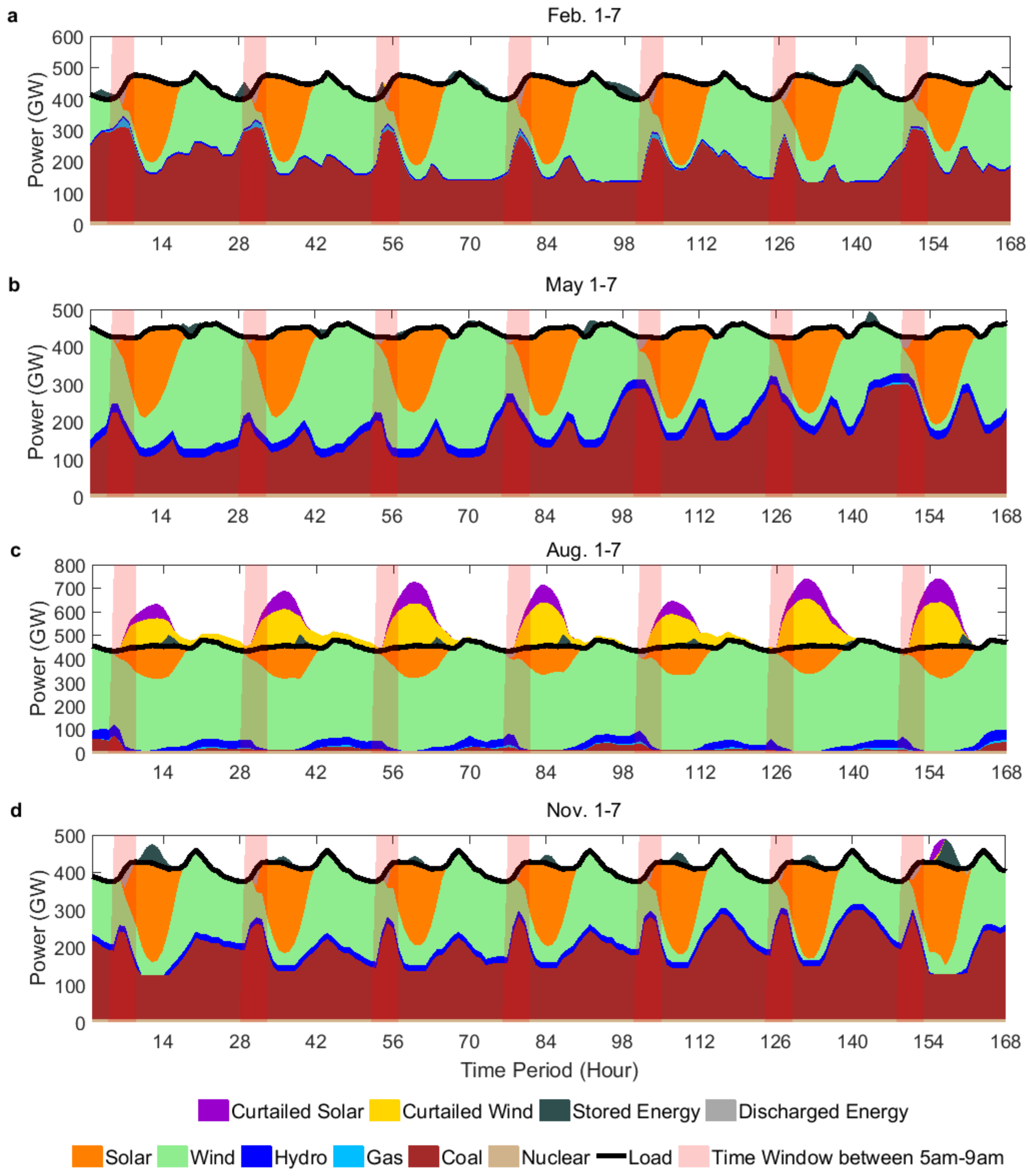


**Supplementary Figure 9 Seasonal hourly power balance.** Hourly power balances defined for seasonally representative weeks on the basis of the 60% renewables standard model simulation: February 1-7 (a), May 1-7 (b), August 1-7 (c) and November 1-7 (d).

**Supplementary Methods: Mathematical formulation of the proposed optimization model**

The decision variables in the operational component are constrained by those in the capacity expansion component mainly in three ways. First, the hourly power generation and hourly online capacities are constrained by the total capacity available for a specific thermal technology (coal or gas):

$$0 \le p_{t,k}^{i} \le \overline{p}_{t,k}^{i} \le \overline{I}_{k}^{i} = I_{k}^{i} + I_{i,k}^{0} \tag{1}$$

where $p_{t,k}^{i}$ and $\overline{p}_{t,k}^{i}$ define the power generation and online capacity for $i^{th}$ category of thermal units in region *k* at time *t*. $\overline{I}_{k}^{i}$, $I_{i,k}^{0}$ and $I_{k}^{i}$ denote the total capacity, the installed capacity and the newly expanded capacity (between 2019 and 2040 in this study) for $i^{th}$ category of thermal units in region *k*.

Second, wind and solar power are constrained by the total capacity, associated with the hourly capacity factor calculated based on physical potential:

$$0 \le p_{t,k}^{w} \le \alpha_{t,k} \cdot \overline{I}_{k}^{w} = \alpha_{t,k} \cdot \left( I_{k}^{w} + I_{w,k}^{0} \right) \tag{2}$$

$$0 \le p_{t,k}^{s} \le \beta_{t,k} \cdot \overline{I}_{k}^{s} = \beta_{t,k} \cdot \left( I_{k}^{s} + I_{s,k}^{0} \right) \tag{3}$$

where $p_{t,k}^{w}$ and $p_{t,k}^{s}$ represent the power generations for wind and solar in region *k* at time *t*. Hourly capacity factors for wind and solar are denoted by $\alpha_{t,k}$ and $\beta_{t,k}$. $\overline{I}_{k}^{x}$, $I_{k}^{x}$ and $I_{x,k}^{0}$ denote the total capacity, the installed capacity and the newly expanded capacity for wind (*x*=*w*) and solar (*x*=*s*) in region *k*.

Third, the hourly power exchange between two regions is constrained by the inter-regional transmission capacity:

$$-L_{j,k} - L_{j,k}^{0} = \underline{L}_{j,k} \le p_{j,k}^{t} \le \overline{L}_{j,k} = L_{j,k} + L_{j,k}^{0} \tag{4}$$

where $p_{j,k}^{t}$ represents the transmitted power between regions *j* and *k* at hour *t*. $\overline{L}_{j,k}$ and $\underline{L}_{j,k}$ denote the maximum and minimum transmission capacity. $L_{j,k}$ and $L_{j,k}^{0}$ denote the installed capacity and the newly expanded capacity for inter-regional transmission corridor *j*-*k*.

The objective of the ESCEM is to minimize the overall system costs, which include two parts: annual operational costs $C_{op}$ with hourly resolution (fuel costs $C_{fuel}$, start-up costs $C_{st}$ and operational costs for storage system $C_{es}$, hydro power $C_h$ and nuclear power $C_{nu}$), and capacity costs $C_{cap}$ (amortized investment costs $C_{inv}$, fixed O&M costs $C_{om}$ and costs for inter-regional network expansion $C_{net}$):

$$\min\ C_{op} + C_{cap} = (C_{fuel} + C_{st} + C_{es} + C_h + C_{nu}) + (C_{inv} + C_{om} + C_{net}) \tag{5}$$

such that:

$$\begin{cases} C_{inv} = \sum_{k=1}^{N_a} \left( \sum_{i=1}^{M_k} a_k^i \cdot I_k^i + a_k^w \cdot I_k^w + a_k^s \cdot I_k^s + \sum_{z=1}^{N_{es}} \left( a_k^{p,z} \cdot \overline{I}_k^{p,z} + a_k^{e,z} \cdot \overline{I}_k^{e,z} \right) \right) \\ C_{om} = \sum_{k=1}^{N_a} \left( \sum_{i=1}^{M_k} f_k^i \cdot \overline{I}_k^i + f_k^w \cdot \overline{I}_k^w + f_k^s \cdot \overline{I}_k^s \right) \\ C_{net} = \sum_{(j,k)\in\Psi} a_{j,k}^l \cdot L_{j,k}^0 \end{cases} \tag{6}$$

$$\begin{cases} C_{fuel} = \sum_{k=1}^{N_a} \sum_{i=1}^{M_k} \sum_{t=1}^{T} c_k^i \cdot p_{t,k}^i \\ C_{st} = \sum_{k=1}^{N_a} \sum_{i=1}^{M_k} \sum_{t=1}^{T} SD_k^i \cdot s_{t,k}^i \\ C_{es} = \sum_{k=1}^{N_a} \sum_{z=1}^{N_{es}} \sum_{t=1}^{T} c_k^{es,z} \cdot \left( p_{t,k}^{dis,z} + p_{t,k}^{ch,z} \right) \\ C_h = \sum_{k=1}^{N_a} \sum_{t=1}^{T} c_k^h \cdot p_{t,k}^h \\ C_{nu} = \sum_{k=1}^{N_a} c_k^{nu} \end{cases} \tag{7}$$

where $N_a$ and $M_k$ denote the numbers of regions and categories of thermal units in region *k*. $a_k^i$ and $f_k^i$ are the amortized investment cost and fixed O&M cost for thermal units. $a_k^x$ and $f_k^x$ are the amortized investment costs and fixed O&M costs for wind (*x*=*w*) and solar (*x*=*s*). $\overline{I}_k^{p,z}$ and $\overline{I}_k^{e,z}$ denote the power and energy capacities for the newly installed storage systems; $a_k^{p,z}$ and $a_k^{e,z}$ represent the corresponding power-specific and energy-specific amortized investment cost for $z^{th}$ category of storages in region *k*. $a_{j,k}^l$ is the investment cost for the newly expanded capacity for inter-regional transmission corridor. $\Psi$ denotes the set of inter-regional transmission corridors. The operational costs for thermal units,

energy storage and hydro power are $c_k^i$, $c_k^{es,z}$ and $c_k^h$. The annual fixed operational cost for nuclear power is denoted as $c_k^{nu}$. $SD_k^i$ represents the start-up cost for thermal units.

Constraints including unit commitment, operations for energy storages, the system power balances and reserves, renewable penetration and intra-regional power flow are considered in the model.

Constraints relating to operations for energy storage are considered based on different regional distributions and storage types. The charging ($p_{t,k}^{ch,z}$) and discharging ($p_{t,k}^{dis,z}$) power for the $z^{th}$ category of energy storage in region *k* is constrained by the non-negative power capacity of newly installed storage:

$$0 \le p_{t,k}^{dis,z} \le \overline{I}_k^{p,z} \tag{8}$$

$$0 \le p_{t,k}^{ch,z} \le \overline{I}_k^{p,z} \tag{9}$$

The energy flow from a storage system is restricted by the following energy balance:

$$e_{t+1,k}^{es,z} = e_{t,k}^{es,z} + \gamma_{es}^{ch,z} \cdot p_{t,k}^{ch,z} - \frac{1}{\gamma_{es}^{dis,z}} \cdot p_{t,k}^{dis,z} - \gamma_{es}^{self,z} \cdot e_{t,k}^{es,z} \tag{10}$$

where $e_{t,k}^{es,z}$ denotes the energy state that satisfies $0 \le e_{t,k}^{es,z} \le \overline{I}_k^{e,z}$. The energy state in the last time interval is equal to that of the first time interval. ($\gamma_{es}^{ch,z}$, $\gamma_{es}^{dis,z}$) and $\gamma_{es}^{self,z}$ represent the energy efficiency and loss (self-discharge) rates.

To quantify the spinning reserve $r_{t,k}^{es,z}$ provided by storage systems, the following formulation is considered:

$$0 \le p_{t,k}^{dis,z} + r_{t,k}^{es,z} \le \overline{I}_k^{p,z} \tag{11}$$

$$0 \le r_{t,k}^{es,z} \le \overline{I}_k^{p,z} \tag{12}$$

$$0 \le e_{t,k}^{es,z} - \frac{1}{\gamma_{es}^{dis,z}} \cdot \left(p_{t,k}^{dis,z} + r_{t,k}^{es,z}\right) - \gamma_{es}^{self,z} \cdot e_{t,k}^{es,z} \tag{13}$$

For chemical storage, the annual energy flow is restricted by the amortized watt-hours passing through the battery:

$$\sum_{t=1}^{T}\left(p_{t,k}^{dis,z} + p_{t,k}^{ch,z}\right) \le \overline{I}_k^{e,z} \cdot H_{es}^{z} \tag{14}$$

where $H_{es}^{z}$ is the annualized watt-hours throughput.

Constraints for the power balance and reserve are considered to meet requirements for the reliability of power grid operation. For every time interval, the power demand equals the sum of the power outputs from all categories of thermal units, hydropower and nuclear power plants, energy storage systems, plus the outputs from wind, solar and inter-regionally transmitted power:

$$\sum_{i=1}^{M_k} p_{t,k}^{i} + p_{t,k}^{w} + p_{t,k}^{s} + p_{t,k}^{h} + p_{t,k}^{nu} + \sum_{j\in\Psi_k} p_{j,k}^{t} + \sum_{z=1}^{N_{es}} \left( p_{t,k}^{dis,z} - p_{t,k}^{ch,z} \right) = D_{t,k} \tag{15}$$

where $\Psi_k$ defines the set of regions connected to region *k*. $D_{t,k}$ denotes the power demand at time *t* for region *k*. $p_{t,k}^{h}$ and $p_{t,k}^{nu}$ represent the hydropower and nuclear power.

The forecasting errors in energy demand $\mathrm{R}^{d}$ and wind $\mathrm{R}^{w}$ and solar $\mathrm{R}^{s}$ output are considered in the following reserve constraint:

$$\begin{aligned} \sum_{i=1}^{M_k} \bar{\mu}_k^{i} \cdot \bar{p}_{t,k}^{i} + \alpha_{t,k} \cdot \bar{I}_k^{w} + \beta_{t,k} \cdot \bar{I}_k^{s} + \sum_{z=1}^{N_{es}} \left( p_{t,k}^{dis,z} + r_{t,k}^{es,z} \right) + \sum_{j\in\Psi_k} p_{j,k}^{t} \\ \geq (1+\mathrm{R}^{d}) \cdot D_{t,k} + \mathrm{R}^{w} \cdot p_{t,k}^{w} + \mathrm{R}^{s} \cdot p_{t,k}^{s} \end{aligned} \tag{16}$$

The constraints of downward reserve are not considered in the model because wind and solar can provide the downward reserve by curtailing their power output in real time during contingency, and thus the downward reserve is not binding in day-ahead scheduling. The minimum load (over generation) problem is captured mainly by the minimum power output and ramping constraints in this formulation.

To meet requirements for system reliability, the system invested capacity must be no less than the required capacity standard:

$$\sum_{i=1}^{M_k} \bar{I}_k^{i} + \lambda_k^{w} \cdot \bar{I}_k^{w} + \lambda_k^{s} \cdot \bar{I}_k^{s} \geq D_k^{\max} \tag{17}$$

where $D_k^{\max}$ is the required capacity to satisfy reliability standards. $\lambda_k^{w}$ and $\lambda_k^{s}$ denote the capacity credits for wind and solar power in region *k*.

Constraints for renewable portfolio requirements are incorporated to set different levels of

renewable penetration for the energy system:

$$\sum_{k=1}^{N_a}\sum_{t=1}^{T}(p_{t,k}^{w}+p_{t,k}^{s})\cdot\Delta t=\Gamma\cdot\sum_{k=1}^{N_a}D_{t,k} \tag{18}$$

where $\Gamma$ ($0 \le \Gamma \le 80\%$ in this study) is the required renewable percentage for the overall renewable portfolio target.

To sum up, the above optimization model minimizes the overall costs for power grid operation and capacity investment under a given renewable penetration, as indicated in equations (5)-(7), constrained by equations (1)-(4) associated with investment and operational decisions, unit commitment, (8)-(14) for storage operation, (15)-(17) for system energy balance and reserves and (18) for the required percentage of renewables. The simulation for India's energy system projected in 2040 covers the timescale of 8760 hours and requires a total of approximately 5 million variables.

**Supplementary Note: $CO_2$ Emissions from energy system**

The total $CO_2$ emissions from India's energy system in 2040 are calculated based on the emission factors of $CO_2$ and the annual power generation of all thermal generators:

$$E^s = e^i \cdot \sum_{k=1}^{N_a} \sum_{i=1}^{M_k} \sum_{t=1}^{T} p_{t,k}^{i,s} \tag{19}$$

where $E^s$ is the total $CO_2$ emissions under scenario $s$. Possible values of $s$ range from 1-5, representing the corresponding scenarios in Supplementary Table 5. $e^i$ denotes the emission factor for $CO_2$ from $i^{th}$ category for thermal units. $p_{t,k}^{i,s}$ defines power generation for $i^{th}$ category for thermal units in region $k$ at time $t$ under scenario $s$.

## References


1. International Energy Agency. Global energy $CO_2$ status report—The latest trends in energy and emissions in 2018. (International Energy Agency, 2019).
2. NASA Land Processes Distributed Active Archive Center (LP DAAC). MCD12C1: Land Cover Type Yearly L3 Global 500 m SIN Grid. https://lpdaac.usgs.gov/dataset_discovery/modis/modis_products_table/mcd12c1 (2012),
3. Central Electricity Authority, Ministry of Power. Coal based Thermal Units Retired Since Sept.'15 to March'18. (Government of India 2018).
4. Central Electricity Authority. Executive summary of Power Sector as on 30-09-2018. (Government of India 2016).
5. Power System Operation Corporation Ltd. Electricity demand pattern analysis. (Power System Operation Corporation Ltd., 2018)
