# Supplementary material for: India’s potential for integrating solar and on- and offshore wind power into its energy system
Source: Nat Commun. 2020 Sep 21;11:4750. doi: 10.1038/s41467-020-18318-7 (PMC7505833; doi:10.1038/s41467-020-18318-7)
Supplement: Supplementary file 3 — Description of Additional Supplementary Files [file 41467_2020_18318_MOESM3_ESM.pdf]

Description of Additional Supplementary Files

File Name: Supplementary Data 1

Description: The supplementary data file contains costs, CO_2 emissions changes and installed capacity information that are derived from output of the cost optimization model used in this paper. The columns denote numerous different scenarios discussed in detail in the manuscript and Supplemental Information at varying renewable penetration levels (from 0 to 80%). The rows correspond to the costs, CO_2 emissions and installed capacity for India in these scenarios at the various renewable penetration levels.
